# JiangShi(僵尸): a widely distributed Mucin-like protein essential for *Drosophila* development

**DOI:** 10.1093/g3journal/jkac126

**Published:** 2022-05-20

**Authors:** Yueping Huang, LingLing Li, Yikang S Rong

**Affiliations:** School of Life Sciences, Sun Yat-sen University, Guangzhou 510275, China; Hengyang College of Medicine, University of South China, Hengyang 421009, China; School of Life Sciences, Sun Yat-sen University, Guangzhou 510275, China; Hengyang College of Medicine, University of South China, Hengyang 421009, China

**Keywords:** mucins and mucin-related proteins, *Drosophila* epithelia, genetic model for mucins

## Abstract

Epithelia exposed to elements of the environment are protected by a mucus barrier in mammals. This barrier also serves to lubricate during organ movements and to mediate substance exchanges between the environmental milieu and internal organs. A major component of the mucus barrier is a class of glycosylated proteins called Mucin. Mucin and mucin-related proteins are widely present in the animal kingdom. Mucin mis-regulation has been reported in many diseases such as cancers and ones involving the digestive and respiratory tracts. Although the biophysical properties of isolated Mucins have been extensively studied, in vivo models remain scarce for the study of their functions and regulations. Here, we characterize the Mucin-like JiangShi protein and its mutations in the fruit fly *Drosophila*. JiangShi is an extracellular glycoprotein with domain features reminiscent of mammalian nonmembranous Mucins, and one of the most widely distributed Mucin-like proteins studied in *Drosophila*. Both loss and over-production of JiangShi lead to terminal defects in adult structures and organismal death. Although the physiological function of JiangShi remains poorly defined, we present a genetically tractable model system for the in vivo studies of Mucin-like molecules.

## Introduction

Epithelial surfaces in animals are in contact with the environment. These surfaces are present in many places, including the respiratory, digestive, and reproductive tracts, as well as body cavities such as the ear, eye, and mouth. In mammals, these surfaces are known to be protected by the mucus barrier or mucosal barrier. The mucus barrier serves physical, chemical, and immunological roles of protection. It also lubricates, such as the role of tears, and mediates substance exchanges (gas, water, etc.) between the environment and internal organs. A major component of the mucus barrier is a class of heavily glycosylated proteins called Mucin. Mucins are commonly mis-regulated in human diseases and their abnormal presence has been used as biomarkers for disease diagnosis (reviewed in Ballester et al. 2019; Bhatia et al. 2019; Hansson 2019; Marimuthu et al. 2021). Therefore, a better understanding of how Mucin’s functions are regulated bears medical significance.

Mucins are large in number, with human having more than 20 different molecular classes (reviewed in Bansil and Turner 2018; Wagner et al. 2018). In addition, Mucin molecules can be quite large, some of them having well over a few thousand amino acid residues (reviewed in Perez-Vilar and Hill 1999; Dekker et al. 2002; Thornton *et al.* 2008). Mucins are abundantly present and some of them have been effectively purified for in-depth studies of their biophysical properties (e.g. Carlstedt et al. 1983; Sheehan and Carlstedt 1984; Carlstedt et al. 1995). On the other hand, there is a limited number of in vivo models for the study of Mucin regulation, with the mouse being the overwhelming choice of model (e.g. Spicer et al. 1995; Velcich et al. 2002; Heazlewood et al. 2008; Roy et al. 2014). In 2014, a model was established for monitoring Mucin physiology in live zebra fish (Jevtov et al. 2014). Therefore, more in vivo models are needed, particularly ones with facile genetics.

Mucins do not share high degree of conservation at the level of primary amino acid sequence. However, different classes of Mucins share unique molecular architectures that are conserved. For example, for nonmembrane associated Mucins, the N- and C-termini are rich in cysteine residues that mediate intermolecular cross-linking between mucin monomers. The central part of these Mucins is rich with proline, threonine, and serine (PTS) residues, making up the PTS-rich domain. Mucins are heavily glycosylated molecules, with some Mucins having 80% of their weight coming from sugar molecules. The Thr and Ser residues in the PTS-rich domain are sites of O-linked glycosylation. Mucins often show N-linked glycosylation as well. Based on these limited but conserved features of Mucins, mucin-like molecules have been identified in a large variety of organisms, including insects (Lang et al. 2007; Schwientek et al. 2007; Syed et al. 2008; Dias et al. 2018), opening up the possibility of introducing new models for Mucin studies.

Mucin-like molecules in *Drosophila* have been preliminarily characterized. In addition, loss-of-function studies have been performed on several Mucin-like proteins in *Drosophila* and other insects (e.g. Garfinkel et al. 1983; Wilkin et al. 2000; Korayem et al. 2004; Syed *et al.* 2012; Reis et al. 2016; Lou et al. 2019; Kim et al. 2020). Nevertheless, the studied molecules so far have either a narrow or unclear tissue distributions that might not be a good representation of general Mucins. In this study, we identified the JiangShi (JS) protein, encoded by the previously uncharacterized *CG14880* gene, as a novel mucin-like molecule broadly produced during *Drosophila* development. JS has a typical “Cys-PTS-Cys” organization of Mucins. It is a secreted glycoprotein and distributes at places where an epithelium–environment interphase exists, consistent with its proposed function as a component of the mucus barrier. Homozygous *js* mutant animals survive to adulthood but die right after eclosion suffering from leakage of bodily fluid from ruptured legs. Interestingly, overexpression of the wild-type protein and its derivatives exerts a dominant negative effect on the endogenous JS functions. JS homologs are readily identified in other insects and crustaceans. We thus identified an additional Mucin-like molecule essential for the development of an insect and established a promising genetic model for the study of Mucins.

## Materials and methods

### *Drosophila* stocks and crosses

Flies were raised on standard cornmeal media and kept at 25°C. The *w^1118^* stock was used as a control. The *js^EY^* stock (BL#16605), a chromosomal deficiency of the *js* locus (*Df(3R)Exel6269*, BL#7736), a *P* transposase insertion at 99B (BL#3664) and a multiply marked chromosome *3* (BL#1783) were obtained from the Bloomington Stock Center of Indiana USA.

#### Cas9-mediated mutagenesis of js

Cas9-induced *js* mutations were obtained using an approach in which the Cas9 enzyme is expressed from the *vasa* promoter in a transgene and the gRNA from the *U6* promoter in another transgene. The gRNA-targeted sequence at the ATG codon of JS is 5′-GAGAACAATCGTCGAAATGT**TGG** (sgRNA-N) and 5′-GGAATCAGGACTATCTGCGC**AGG** (sgRNA-C) at the STOP codon (the PAM sequences in bold). All mutations were verified by PCR amplification followed by sequencing using DNA from homozygous flies as templates. The mutant sequences are provided in the *Sequencing Results* in the Supplementary Material.

#### Mobilization of the P element in js^1^

The *js^1^* allele is associated with a *white^+^*-marked *P* element, which produces pigmented eyes in an otherwise *white*-mutant background. We introduced a third chromosome transposase source (*Δ2-3*) into *js^1^* heterozygous flies and recovered white-eyed progeny carrying the original *js^1^* chromosome *3*. These chromosomes were made homozygous and checked for the status of the recessive js phenotype. Multiple independent excision events (>5) that restored the js phenotype were sequenced and all had precise excision of the P element. Several white-eyed events retain the recessive js phenotype. They were subject to genomic PCR aimed at amplifying across the insertional site of the original P element. One such allele, *js^3-1^*, yielded a PCR product of about 7.5 kb. Sequencing revealed the presence of a part of the original P element (sequences are provided in the *Sequencing Results* in the Supplementary Material). The other events did not yield PCR products presumably because of the larger size of the remaining P element fragment making the PCR inefficient.

#### Complementation tests of js alleles

To map the original *js^1^* allele, a multiply marked third chromosome was used to map the *white^+^* marker between *cu* and *e*, and close to *Sb* subsequently. Chromosome deficiencies of the regions were used in complementation tests further narrowing the range to about 10 genes. Genomic PCR with primers 1 kb apart covering the entire region was performed using DNA from *js^1^* homozygotes as template. Elongation time was limited so that a large insertion between the primers, i.e. the *P* element, would not yield a PCR product. This PCR test tentatively pinpointed the P element insertion site at the first exon of *CG14880*, which was later confirmed by PCR and sequencing of imprecise excision events induced by P transposase.

To conduct pairwise complementation tests among *js* alleles (*js^1^*, *js^3-1^*, *js^EY^*^,^*js^cas-1^*, *js^cas-2^*, *js^c-term^*, *df*), 5–8 pairs of *js* heterozygotes carrying 2 different alleles were mated and transferred to a new vial after 5 days. They were discarded 10 days after they were initially crossed. Progeny were scored for 10 days after they started to emerge. Typically, more than 200 progenies emerged from each cross and no survivors were *js* mutants. Between 10% and 60% of the *js* mutants did eclose but soon died and displayed the js phenotype (for details see *Results*), and they were not counted as survivors.

### Plasmid construction

For Cas9 mediated tagging at the C-terminus of JS, a ∼2-kb fragment centered at the STOP codon (from 948 bp 5′ of “T” of the TAG codon to 1103 bp 3′ of “G”) was subcloned from genomic DNA and a DNA fragment encoding the 3 fluorescent proteins (mCherry, dsRED, and EGFP) was individually inserted just upstream of the STOP codon using bacterial recombineering (Zhang *et al.* 2014). These plasmids served as donor DNA in homologous recombination in which the 2 DNA fragments flanking the fluorescent gene are homologous to each side of the Cas9-induced DNA break on the chromosome. This donor plasmid was injected into flies expressing Cas9 and a gRNA targeting the STOP codon of *js* (sgRNA-C). Knock-in events were screened by PCR and verified by genomic PCR followed by sequencing. Sequences are provided in the *Sequencing Results* in the Supplementary Material.

For JS overexpression, full-length *js* cDNA was amplified from cDNAs reverse transcribed from RNA isolated from wild-type adults. The cDNA was cloned into the multiple cloning sites of pUASTattB generating the *UAS-js* construct for subsequent phiC31-mediated integration at the 75B genomic location. A fragment encoding dsRED was inserted into *UAS-js* just before the STOP codon of JS by recombineering generating the *UAS-js-dsRED* construct. Based on *UAS-js-dsRED*, 2 small deletions were introduced individually by site-directed mutagenesis generating the *UAS-ΔCBD-I* and -*II* constructs. All constructs were confirmed by sequencing. Sequences are provided in the *Sequencing Results* in the Supplementary Material.

### Western blot reagents and assays

A corresponding DNA fragment encoding the JS antigen was subcloned from cDNA into pET28a for expression. Bacterial expression was induced with 0.1 mM IPTG, and the recombinant protein was purified as inclusion bodies. Briefly, inclusion bodies were separated from soluble fractions by centrifugation. Pellets were washed 3 times with 2 M urea and 1% Triton X-100, and 3 times with 2 M urea alone. Each wash constituted a brief sonication of the pellet in wash buffer followed by centrifugation and disposal of the supernatant. The washed pellet was dissolved in 8 M urea and used in the immunization of rabbits to generate polyclonal anti-sera. Rabbit anti-sera were used at a dilution of 1:5,000 for western blotting. The transfer of JS protein onto PVDF membrane was done by a protocol developed for studying Mucin-D (Kramerov et al. 1996, 1997). Briefly, extracts were prepared with traditional SDS loading buffer. SDS-PAGE was run with the standard 1XTGS (Tris-Glycine-SDS) buffer at a Voltage of 100 V and transferred in 10 mM sodium borate buffer (pH 9.2) for 3 h at 0.4 A on ice. The Wheat Germ Agglutinin (WGA) assay was performed as described (Fujioka et al. 2018), except using the transferring conditions described above.

### Microscopy

Life imaging of fluorescently labeled JS proteins produced from *js* knock-in alleles was performed on a Zeiss Image2 fluorescence microscope. Embryos were mounted in 50% glycerol and observed directly. Larvae were mounted in 50% glycerol, heated on a 65 °C heating block for a few seconds till they stopped moving, and covered with a coverslip for microscope observation. We confirmed that while this heat-induced immobilization facilitates life imaging, it does not alter the expression pattern nor intensity of the fluorescently tagged JS proteins. Pupae and adults were mounted in glycerol and observed directly.

For immunostaining, anti-mCherry antibody from Abcam (ab167453) and anti-dsRED from Santa Cruz (sc-101526) were used at various dilutions but did not produce satisfactory results.

Transmission electronic microscope (TEM) and scanning electronic microscope (SEM) analyses were performed at the core facility of the School of Life Sciences Sun Yat-sen University China, following standard protocols. For TEM analyses, wild-type (*w^1118^*) and *js* mutant tissues were prepared as follows: legs were dissected from adults within 1 day after eclosion. Gut, pericardial cells, and trachea were dissected from larvae in the third instar. Tissues were fixed overnight in 4% glutaraldehyde at 4°C. After 3 washes with 0.1 M sodium cacodylate (pH 7.2), tissues were stained with 1% osmium tetroxide for 1 hr at room temperature. They were washed 3 times again and stained with uranyl acetate overnight. After a standard ethanol dehydration series (5 min each in 25%, 50%, 75%, 95% EtOH, and 3 × 10 min in 100% anhydrous ETOH), tissues were rinsed in propylene oxide twice before they were embedded using standard procedures. Thin sections (100 nm) were cut and collected on support grids and stained with uranyl acetate for 15 min, followed with 10 min in lead citrate. Micrographs were taken at 120 kV on a JEM-1400 TEM. For SEM analyses, legs were dissected from adults within 1 day after eclosion, fixed for overnight in 4% glutaraldehyde at 4°C, washed 3 washes with PBS (pH 7.0), dehydrated with a graded ethanol series (5 min each in 25%, 50%, 75%, 95% EtOH, and 3 × 10 min in 100% anhydrous ETOH). They underwent the processes of critical point drying and metallizing before micrographs were taken on a Hitachi S-3400N microscope.

### Silver nitrate feeding

Feeding was performed as described previously (Zhang, Zhao, Chao, *et al.* 2013). Briefly, first instar larvae of *js^1/1^* and *js^1^/TM6B* were selected, placed separately on agar-only plates supplemented with regular yeast paste or yeast paste containing AgNO3 (2.0 g yeast in 3.5 ml 0.005% AgNO3 solution) and allowed to develop at 25°C until adulthood. Pupae from both genotypes were skinned for photography.

## Results

### The Jingshi phenotype

**Fig. 1.**
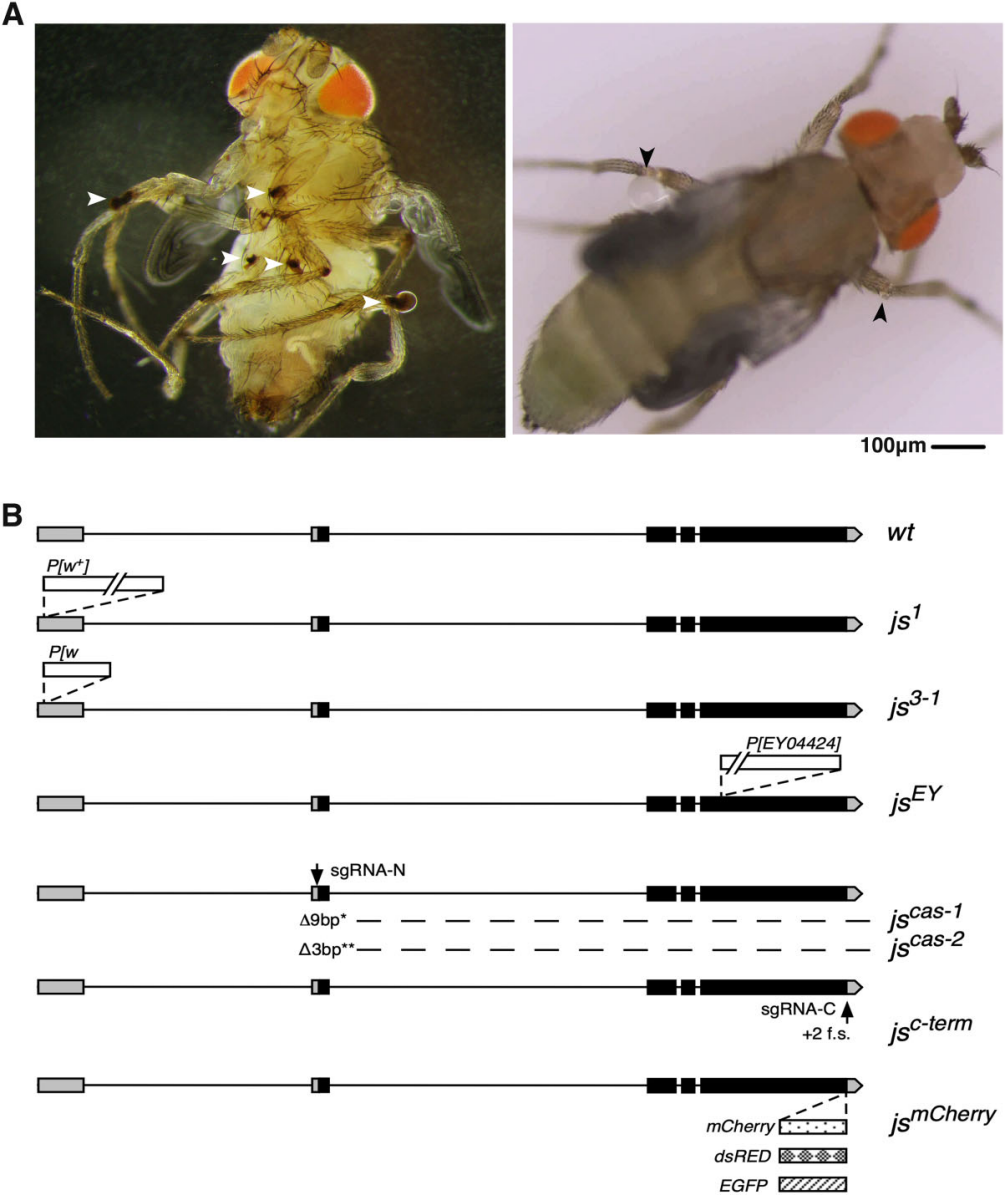
The phenotype and genomic structure of *js* alleles. a) Morphological defects of *js* mutant adults. At the left is a darkfield image of a *js^1/df^* adult showing blackish substance at multiple leg-joints indicated with white arrowheads. At the right is a brightfield image showing liquid droplets forming at the leg-joints (arrowheads) of a *js^1/df^* adult. b) Diagrams of the *js* locus. The genomic structure of the wild-type (*wt*) *js* locus is shown at the top with exons shown as boxes and introns as lines. Noncoding exons are shaded in grey and coding ones in black. The arrow indicates the direction of transcription. The *js^1^* allele has a *white^+^* (*w^+^*) marked *P* element inserted at the first but noncoding exon so that *js^1^* flies have pigmented eyes. The *js^3-1^* allele has a ∼7-kb fragment of the original *P* element remained in which a part of the *w^+^* gene was deleted so that *js^3-1^* flies are white-eyed. The *js^EY^* allele has a *P* element inserted in the largest coding exon of *js*. The *js^cas-1^* (a 9-bp deletion) and *js^cas-2^* (a 3-bp deletion) mutations were generated by CRISPR/Cas9 mediated mutagenesis utilizing the “sgRNA-N” guide RNA, targeting the START codon of *js* (marked with an arrow). The *js^c-term^* alleles are +2 frame shift (f.s.) mutations induced by CRISPR/Cas9 utilizing the “sgRNA-C” guide RNA, marked with an arrow. The *js^mCherry^* knock-in allele has a DNA fragment encoding mCherry inserted at the endogenous *js* locus just upstream of the STOP codon. Similarly, a fragment encoding dsRED or EGFP was knocked into *js* at the identical position.

We fortuitously recovered a recessive mutation on the third chromosome that can only be kept as a heterozygous stock, and homozygotes display the following novel phenotypes. They develop into pharate adults and the majority eclose successfully. However, adults emerged with weak legs and showed uncoordinated movements, hence the Chinese name *jiangshi* (*js*): a walking dead. Leg joints on all of the adults carry dark colored and sticky substances (Fig. 1a). These adults ultimately drowned in food or were stuck to the side of the vials, likely due to the blackish substances coming from the joints. The description of the *jiangshi* phenotype matches very well with that of a previously isolated but nonextant *arthritics* (*arth*) mutation. Without a better description of the *js* phenotype, we use that for the *arth* mutation verbatim: “legs weak with pigmented joints; tarsal segments frequently askew with claws fused; movements somewhat uncoordinated; brownish-black pigment present at joints …; most frequently in meso- and metathoracic legs between femur and tibia but sometimes between coxa and trochanter or proximal to coxa” (flybase.org). Since *arth* has been mapped to the *X* chromosome (Fleming et al. 1989), *arth* and *js* are 2 different genes.

A video recording the eclosion of a *js* adult (Supplementary Movie 1) clearly shows that fluids form droplets of various sizes on leg joints after the fly emerges from the pupal case (see also Fig. 1a). We therefore postulate that leg joints of *js* adults rupture during the strenuous eclosion process, letting out body fluids that form the basis for the blackish substances. The change from colorless droplets to blackish substances might be related to the melanization process of *Drosophila* hemolymph. We did not further investigate the nature of the black matter but chose instead to focus on uncovering the genetic cause for this unique phenotype.

### The *js* gene

The original *js* allele, which we name *js^1^*, is associated with a *white^+^* eye marker. Using a combination of recombination-based and chromosomal deficiency-based mapping methods, followed by PCR and genomic sequencing, we determined that *js^1^* is associated with a *P* transposable element inserted into the 5′ *UTR* of the gene *CG14880* (for mapping details see *Materials and Methods*). Figure 1b shows the genomic structure of the *js* locus and its various alleles. Several lines of evidence support that mutations in *CG14880* cause the *js* phenotype. First, we mobilized the *P* element in the germline by expressing *P* transposase and recovered events of precise excision of the element that reverses the mutant phenotype (see *Materials and Methods*). We also recovered imprecise excision events losing only part of the *P* element but retaining the phenotypes (the *js^3-1^* allele). Second, an independent *P* element insertion into one of the coding exons of *CG14880* causes the recessive js phenotype (the *js^EY^* allele). Third, we recovered 2 CRISPR/Cas9-induced small deletions of the start code of *CG14880*. These mutations also cause the js phenotype (the *js^cas-1^* and *js^cas-2^* alleles). Fourth, we used CRISPR/Cas9 to make mutations around the Stop codon of *CG14880* and recovered multiple alleles deleting the Stop codon while causing a + 2 frameshift, which displayed the js phenotype (the *js^C-term^* allele). Lastly, all of the above point mutations of *js*, when trans-heterozygous with each other or with a chromosomal deficiency that deletes the *CG14880* region (*Df(3R)Exel6269*), produced the js phenotype (see *Materials and Methods* for details of the complementation tests). Therefore, our extensive genetic analyses establish that mutations in *CG14880* are responsible for the js phenotype.

### JS protein has features of Mucins

**Fig. 2.**
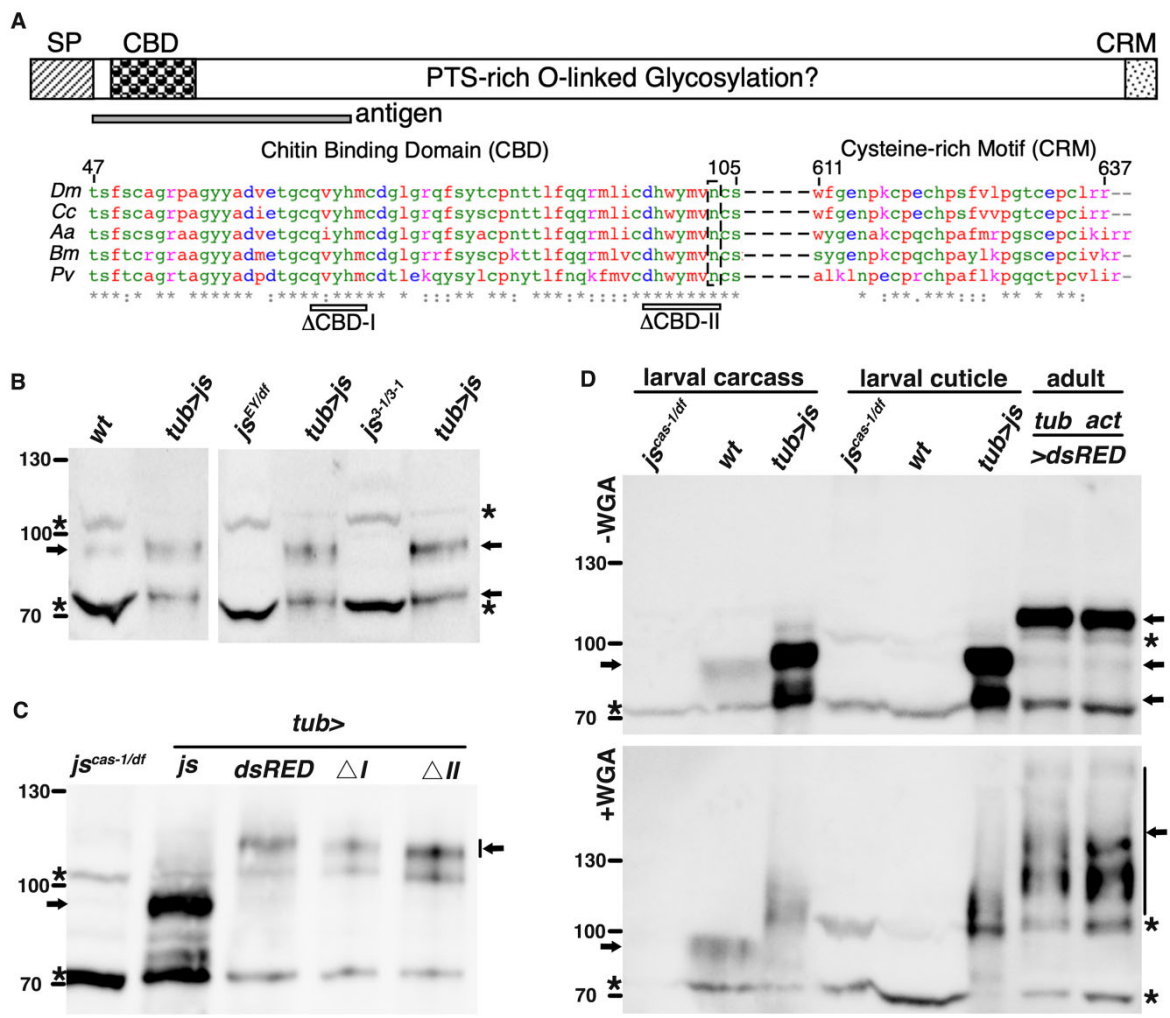
Features of the JS protein and the detection of its glycosylation. a) A diagram of the structural elements of the JS protein is presented at the top: SP (signal peptide); CBD; PTS-rich domain; and CRM. The antigen used for raising antibodies is indicated beneath the domain diagram. Sequence alignments, generated by clustal omega, for the CBD and CRM domains are shown for the following species: *Dm* (*Drosophila melanogaster*, NP_650538.1), *Cc* (*Ceratis capitata*, XP_012157262.1), *Aa* (*Aedes aegypti*, EAT42245.1), *Bm* (*Bombyx mori*, XP_004927149.1), and *Pv* (*Penaeus vannamei*, pacific white shrimp, XP_027211762.1). At the top of the alignment, the residue numbers are provided for the *Drosophila* protein. Beneath the alignment, the ranges of the 2 CBD deletions are shown for I (deletion of 5 residues) and II (deletion of 7 residues). The N-linked glycosylation site is marked with a black box. b) Western blot detection of JS protein in adult tissues. Whole extracts from wild type (*wt*), 2 *js* mutants and adults overexpressing full-length JS from the *tubulin* Gal4 driver (*tub>js*) were used for western blotting. The overexpressing extracts were loaded at a 1:50 dilution. The JS protein bands are indicated with arrows. Nonspecific bands, marked with asterisks, were used as loading controls. Note that one of the JS bands (∼70 kDa) in the overexpression extracts ran close to one of the major nonspecific bands. Markers, with sizes indicated in kDa, are shown to the left. c) Western blot detection in adults overexpressing various forms of the JS protein. Extracts from flies overexpressing various derivatives of the JS protein were used along with extracts from *js* mutant extract. The overexpression extracts were used at a 1:50 dilution. Overexpression was driven by the tubulin Gal4 driver (tub>). *js*: full-length JS overexpression; *dsRED*: full-length JS tagged with dsRED at the C-terminus; *ΔI*: CBD-I deleted JS tagged with dsRED at the C-terminus; *ΔII*: CBD-II deleted JS tagged with dsRED at the C-terminus. The JS protein bands are indicated with arrows. Nonspecific bands are marked with asterisks. Markers, with sizes indicated in kDa, are shown to the left. d) Western blot-based WGA assay for detection of JS protein and its glycosylation state. Extracts were derived from larval cuticles (middle 3 lanes), larval carcasses devoid of cuticles (left 3 lanes) and whole adults (right 2 lanes). Larval extracts were from *js* mutants, wild type, or animals overexpressing full-length JS driven by tubulin Gal4 (*tub>js*). Adult extracts were from animals overexpressing full-length JS tagged with dsRED, driven by tubulin (tub) or actin5C (act) Gal4 driver. The overexpression extracts were used at a 1:10 dilution. Bands corresponding to JS are indicated by arrows, while nonspecific bands with asterisks. Extracts were run in parallel on 2 gels, the lower of which contained WGA (+WGA). Markers, with sizes in kDa, are indicated to the left.

The *js* gene encodes a protein of 637 residues (Fig. 2a). The first 30 residues of JS are predicted to be a signal peptide suggesting that JS is a secreted protein (Supplementary Fig. 1). C-terminal to the signal peptide, starting around the 51st residue, is a domain of about 60 residues that has been annotated as a chitin-binding domain (CBD). The CBD domain is highly enriched with conserved cysteine residues (∼10%). Therefore, mature JS protein has a Cys-rich N-terminus. At the very C-terminus of JS, we identified a conserved motif (about 20 residues in length) also enriched with cysteines (∼20%), which we named cysteine-rich motif (CRM). Between CBD and CRM lies the majority of the JS residues, which are noticeably enriched with PTS residues (PTS-rich): 175 of the 510 intervening residues (34%). PTS domains of mammalian Mucins are modified with O-linked glycosylation, while the presence of N-linked glycosylation is also common. In JS, 2 N-linked glycosylation sites have been identified (Baycin-Hizal et al. 2011), with 1 inside CBD (Fig. 2a). The above features that we described for JS: a secreted protein with 2 Cys-rich domains flanking a PTS-rich region, are consistent with the basic domain organization of nonmembranous Mucin-like molecules previously identified in various vertebrates. However, we note that JS was not identified in prior bioinformatic studies of insect Mucin-like molecules (e.g. Lang et al. 2007; Syed et al. 2008). We note that clear JS homologs based on sequence homology are present in insects and crustaceans (Fig. 2a and Supplementary Fig. 2).

An essential feature of Mucins is O-linked glycosylation. To investigate whether JS is glycosylated, we generated antibodies against an antigen consisting of the first 198 residues of the mature JS protein (Fig. 2a). In normal western blots (several shown in Fig. 2), we observed a band of about 95KD in size. The signal for this band is weak and variable, and it is different from JS’s predicted size of 70KD, but it is consistently missing in *js*-mutant extracts. We reasoned that the weak JS signals on a western blot could have two possible but mutually exclusive causes. First, the level of JS might be relatively low. Second, our antibodies, raised against a bacterially expressed JS antigen, might be inefficient in recognizing the endogenous JS proteins especially considering that JS is likely a glycosylated protein with a potentially high propensity to form intra- and intermolecular disulfide bonds. We note that the JS antigen encompasses a site where the endogenous JS protein is modified by N-linked glycosylated (Fig. 2a). To gain more confidence on the specificity of our antibodies, we overexpressed JS using a construct that places *js* cDNA encoding the full-length protein as well as its various derivatives under the control of the Gal4 activator. We used either a *tubulin* (*tub*) or an *actin5C* (*act*) Gal4 driver to achieve ubiquitous overexpression. As shown in Fig. 2, b–d, our antibodies very strongly recognize a dominant band of about 95 kDa and a minor one of about 70 kDa specifically under the condition of JS overexpression, suggesting that both protein species are produced from the JS-overexpressing transgene and further validating our antibodies. Note that the overexpressing extracts were loaded at much-diluted amounts (1:10 to 1:50). We postulate that the size increase of the endogenous JS protein is related to JS being a putative glycoprotein.

The traditional method of using de-glycosylases (Roth et al. 2012) proved unproductive in characterizing JS’s state of glycosylation. We employed a gel electrophoretic method that cleverly takes advantage of the ability of WGA to bind and retard the mobility of glycoproteins in traditional SDS PAGE gels (Kubota et al. 2017). As shown in Fig. 2d, the endogenous and particularly the over-expressed JS display mobility retardation in a WGA gel when compared with one without WGA, and when markers and nonspecific bands were used as internal controls for protein mobility. Interestingly, both the 70 and 95-kDa bands from the JS-overexpressed samples showed WGA-retarded mobilities, suggesting both are glycosylated but perhaps to different extents. Therefore, how the 2 protein species differ in mobility cannot simply be due to the absence or presence of glycosylation. Interestingly, overexpressed JS is abundantly present in extracts made from cuticles of third instar larvae (Fig. 2d), consistent with JS being a secreted protein. In summary, JS protein bears common features of Mucin-like molecules with the important presence of glycosylation.

We note that WGA is often used for the detection of *N*-acetyl-d-glucosamine (O-GlcNAc), while mammalian mucins are enriched with *N*-acetyl-galactosamine (O-GalNAc). Therefore, although our results support JS being a glycoprotein, whether it possesses O-GalNAc modifications requires further investigations.

### JS is a secreted protein highly enriched at epithelia and widely expressed in development

Although our antibodies were able to recognize the JS protein on western blots, they failed to generate satisfactory results in immunostaining experiments using JS nonoverexpressing tissues. To study the localization of endogenous JS, we generated a knock-in allele of *js* by CRIPSR/Cas9-mediated homologous recombination, in which a DNA fragment encoding the mCherry fluorescent protein was placed in-frame before the STOP codon (*js^mCherry^*, Fig. 1b). The mCherry tag does not disrupt JS functions as *js^mCherry^* homozygous flies are viable, fertile, and kept as a homozygous stock. Thus, the fluorescence from mCherry allows us to deduce the normal localization of endogenous JS proteins. To minimize interference from auto-fluorescence, we only identify red signals as originated from the JS-mCherry protein when the same region does not emit green/yellow fluorescence, and when the same region does not emit red fluorescence under the wild-type background (Supplementary Fig. 3). We present below, to the best of our abilities, a comprehensive description of JS-mCherry distribution in development.

#### Early developmental stages

**Fig. 3.**
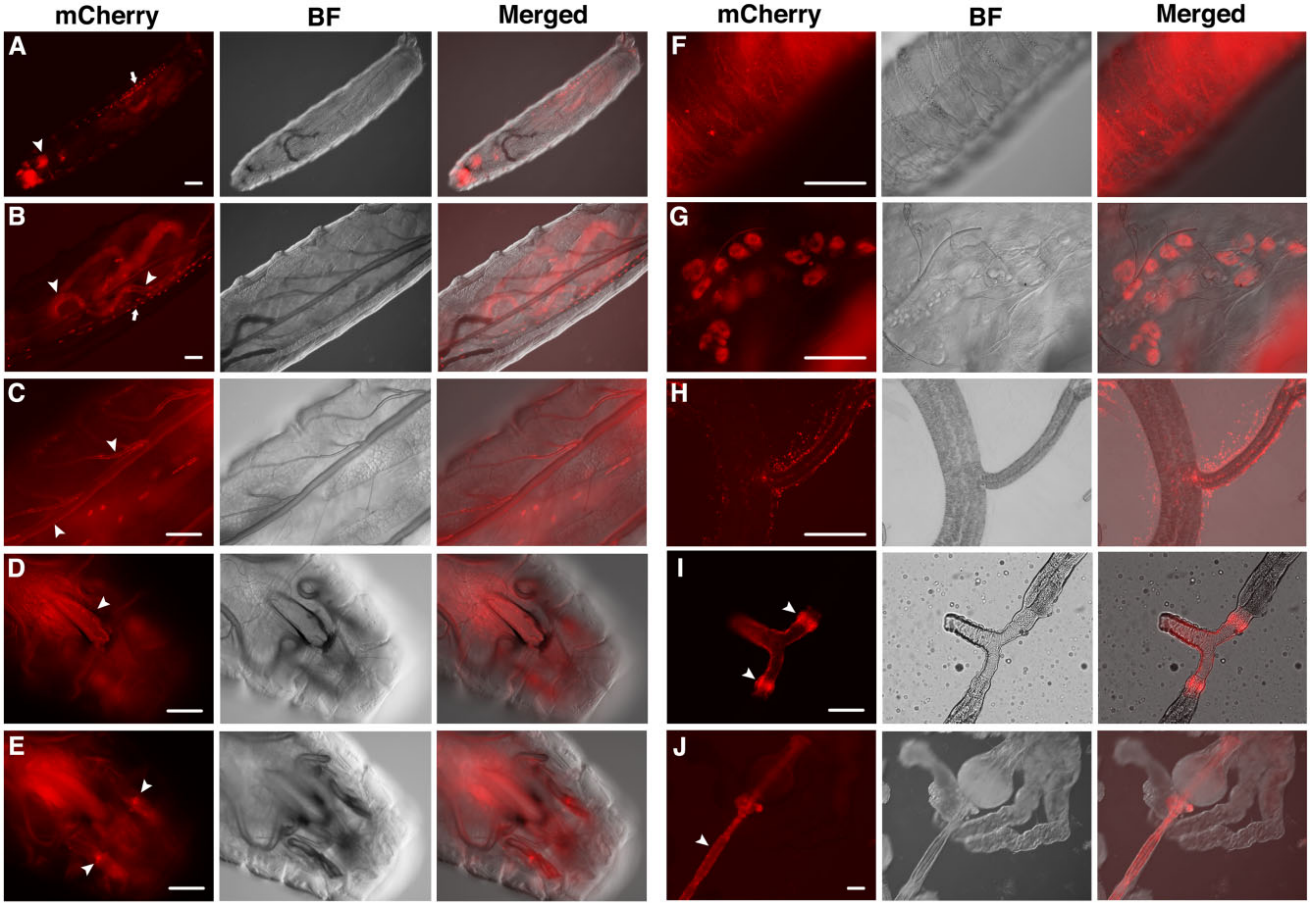
JS localization during larval development. Live images are provided as triplets: red fluorescence (mCherry), brightfield (BF) and the merged of the 2. a) A whole mount first instar larva. Arrow indicates pericardial cells, and arrowhead indicates the mouth area. b) A second instar larva. Arrow indicates pericardial cells, and arrowheads indicate guts. c) A third instar larva. Arrowheads indicate trachea. d and e) Mouth area of a third instar larva. Arrowhead in (d) indicates the pharynx. Those in (e) indicate the humerus discs. f) Cuticular view of a first instar larva. g) Garland cells from a third instar larva. h) JS puncta surrounding trachea from a third instar larva. i) duct area of a dissected salivary gland from a third instar larva. Arrowheads indicate the imaginal rings. j) dissected foregut (arrowhead) from a third instar larva. Scale bars indicate 100 µm.

The mCherry signal is first visible around twelve hours after egg laying but become prominent in first instar larvae (Fig. 3a), showing prominent fluorescence at the mouthpart, pharynx, and the pericardial cells. Interestingly, right after hatching, JS-mCherry signals are visible throughout the larval cuticle and in the form of puncta (Fig. 3f).

JS distribution does not change significantly as the larva grows. JS-mCherry signal is present at: guts (Fig. 3, b and j), trachea (Fig. 3, c and h), pharynx (Fig. 3d), humerus discs (Fig. 3e), pericardial, and garland cells (Fig. 3, b and g). We observe JS signals surrounding the individual ducts and imaginal rings of the salivary glands (Fig. 3i).

#### JS-mCherry molecules form puncta

**Fig. 4.**
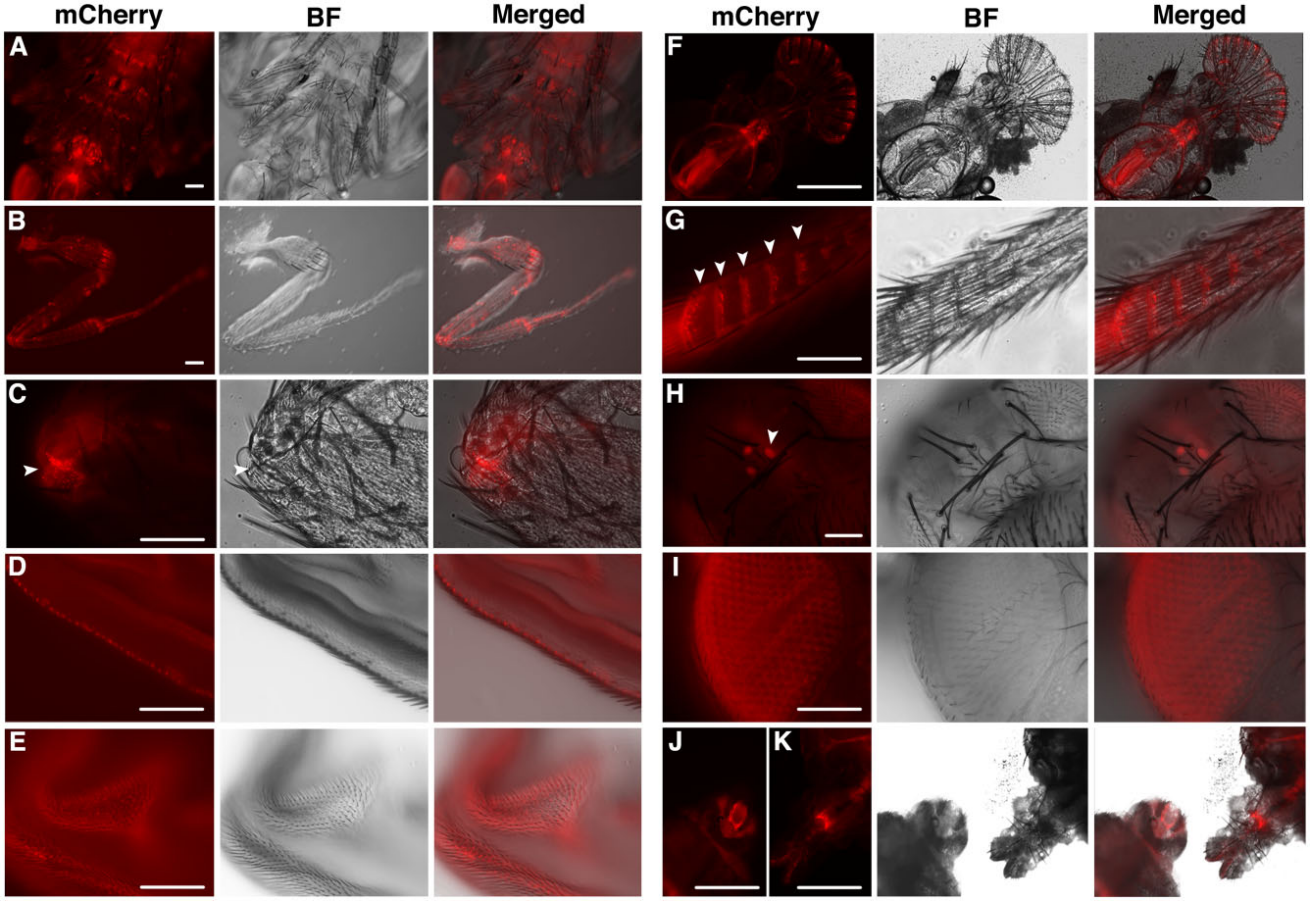
JS localization in pupal and adult stages. a) A late-stage pupa showing mCherry signals in multiple leg joints. b) A dissected front leg from an adult. c) A closeup picture of an adult leg joint showing JS puncta surrounding the junction (arrowhead). d) Wing blade from an adult showing a row of JS puncta. e) Surface view of a pupal wing showing JS puncta. f) Mouth area of an adult. g) A closeup view showing rows of JS puncta (arrowheads) aligning with the rows of bristles on an adult leg. h) JS puncta in the 3 ocelli (arrowhead). i) Surface view of an adult eye showing JS puncta surrounding each ommatidium. j) A female genital. k) A male genital. Scale bars indicate 100 µm.

We notice that JS-mCherry signals appear as puncta in various tissues (Figs. 3 and 4). This focused appearance seems to be inconsistent with the expected distribution of Mucin-like proteins, which would be a more even distribution over the entire epithelium. However, one cannot rule out that these puncta only represent some of the JS molecules since our detection method depends on mCherry’s ability to fluoresce in the different chemical environments that JS-mCherry exists in. It is possible that some of the JS-mCherry molecules do not fluoresce. Results described in the next section lend support to this proposition.

We generated other *js* knock-in alleles in which either a dsRED or an EGFP tag was placed at the C-terminus of JS (Fig. 1b), similarly to the mCherry tag. Interestingly, none of these 2 alleles, although allowing homozygous flies to survive and reproduce, displays fluorescence similar in pattern or intensity to that of JS-mCherry, suggesting that the natural environment where JS resides is less permissive for dsRED or EGFP fluorescence. The same environment seems to be more permissive to mCherry fluorescence, although not necessary for all of the JS-mCherry populations. Therefore, it cannot be ruled out that JS-mCherry puncta that we observed represent 1 class of the JS molecules, perhaps as intermediates in the process of JS secretion or maturation, and that the functioning JS-mCherry molecules at the epithelia might not be fluorescently visible. In other words, we cannot be confident that most of the JS-mCherry molecules have been visually localized in our live analyses. Unfortunately, immunostaining with anti-mCherry and anti-dsRED antibodies using *js^mCherry^* and *js^dsRED^* tissues, respectively, did not yield satisfactory results.

#### Pupal and adult stages

JS-mCherry is abundant in all of the major joints of legs, consistent with the most prominent phenotype of *js* mutant adults (Fig. 4, a–c and g). It is also highly significant on wing (Fig. 4, d and e), the mouth (Fig. 4f), and genital of both sexes (Fig. 4, j and k). JS-mCherry punctum seems to be located at the base of each major bristle. This is most prominent for bristles on the legs and wings, possibly due to that those areas are under less interference from internal fluorescence (Fig. 4, d–g). Remarkably, we observed puncta of JS-mCherry surrounding each ommatidium in the eye and this pattern is reproducible from GFP fluorescence present in *js^egfp^* knock-in flies (Fig. 4i and Supplementary Fig. 4). In ocelli of both *js^mCherry^* and *js^egfp^* flies, we also observed strong JS puncta (Fig. 4h and Supplementary Fig. 4).

### Potential functions of JS

**Fig. 5.**
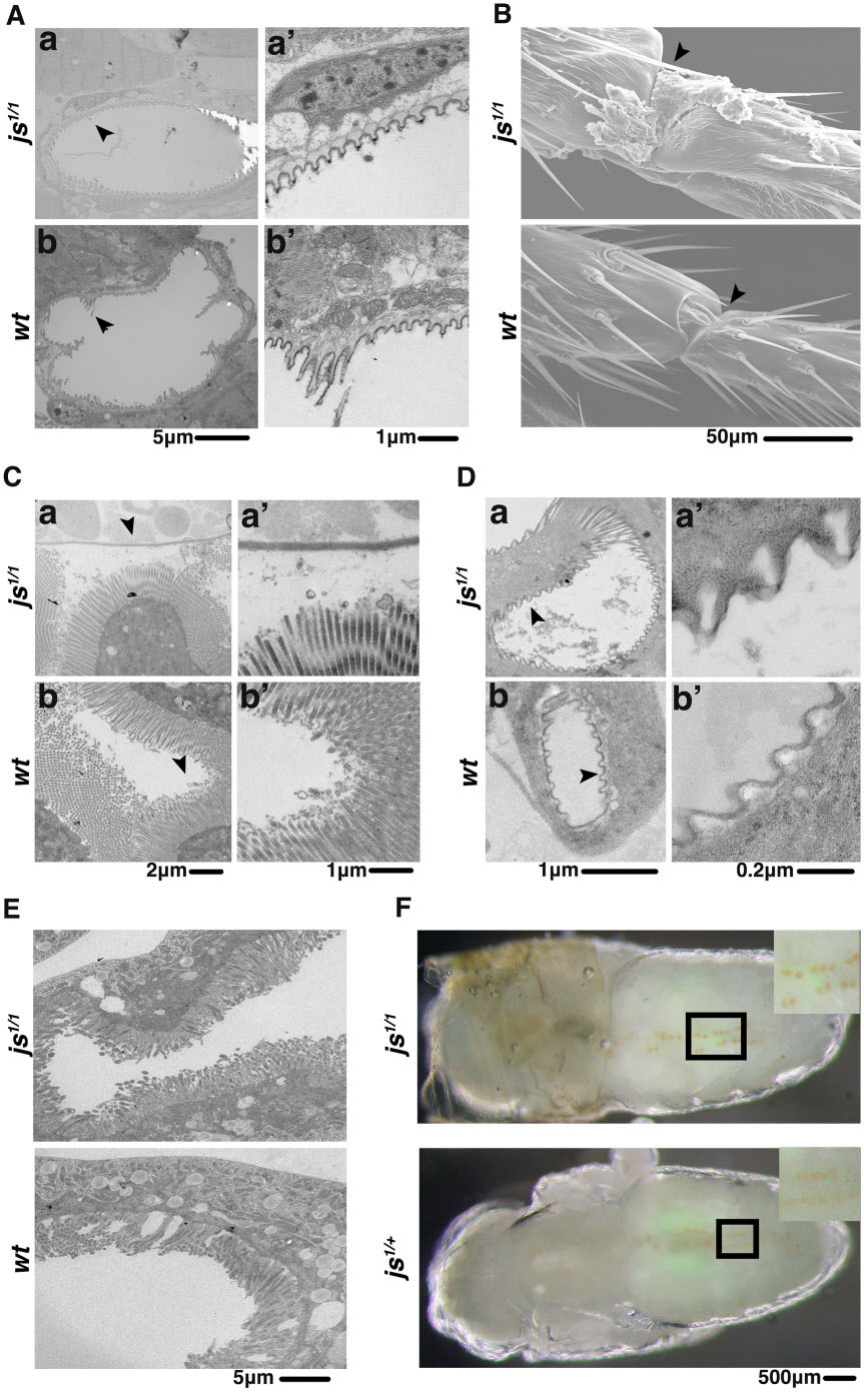
Ultrastructural studies of *js* mutants. For all images, genotypes are listed to the left. A) TEM images of leg cross-sections. The area marked with an arrow in (Aa) and (Ab) was photographed again but with a higher magnification and shown in (Aa′) and (Ab′), respectively. B) SEM images of leg joints, with arrowheads demarcating the area where extra substances are visible covering the joint from the mutant but not wild type. C) TEM images of cross sections of larval guts. The area marked with an arrow in (Ca) and (Cb) was photographed again but with a higher magnification, and shown in (Ca′) and (Cb′), respectively. D) TEM images of cross sections of larval trachea. The area marked with an arrow in (Da) and (Db) was photographed again but with a higher magnification, and shown in (Da′) and (Db′), respectively. E) TEM images of cross sections of nephrocytes (pericardial cells). F) Bright field images of pupae showing the accumulation of AgNO3 in the nephrocytes, with the area marked with a box shown at a higher magnification at the top right corner.

To shed lights on possible functions of JS, we conducted electron microscopy (EM) imaging of tissues where JS is present. SEM reveals “extra” substances covering mutant leg joints, likely corresponding to the blackish substance under visible lights (Fig. 5B). However, TEM imaging of cross-sections of legs failed to uncover apparent structural differences between wild-type and *js* mutants (Fig. 5A). Similarly, cross-sectioning of guts (Fig. 5C) or trachea (Fig. 5D) did not reveal abnormalities in the mutant tissues.

One of the most consistent tissues where JS is present is the pericardial cells (Fig. 3). Nevertheless, cross-sectioning of larval pericardial cells did not reveal structural abnormalities suggestive of a functional loss in these cells (Fig. 5E). Pericardial cells serve to filter the hemolymph, similar in function as mammalian nephrocytes (reviewed in Helmstädter and Simons 2017). Disruption of nephrological function in *Drosophila* has been reported to cause sensitivity to silver poisoning (Weavers et al. 2009; Zhang, Zhao, Chao, *et al.* 2013; Ivy et al. 2015). We therefore treated flies with silver nitrate (AgNO3) but did not observe an overt effect on the development of *js* mutants in that homozygous larvae fed with AgNO3 were able to eclose as adults. In addition, bright field imaging clearly shows that silver salts were successfully retained in larval nephrocytes in *js* mutant larvae (Fig. 5F).

In summary, our ultra-structural studies reveal largely normal tissue morphology in *js* mutant animals leaving the potential physiological function of JS undefined.

### Overproduction disrupts JS function

Although all *js* mutant alleles behaves as recessive mutations, we discovered that overexpression of the wild-type JS protein is sufficient to produce very similar if not identical phenotypes to those in recessive mutants described previously.

**Fig. 6.**
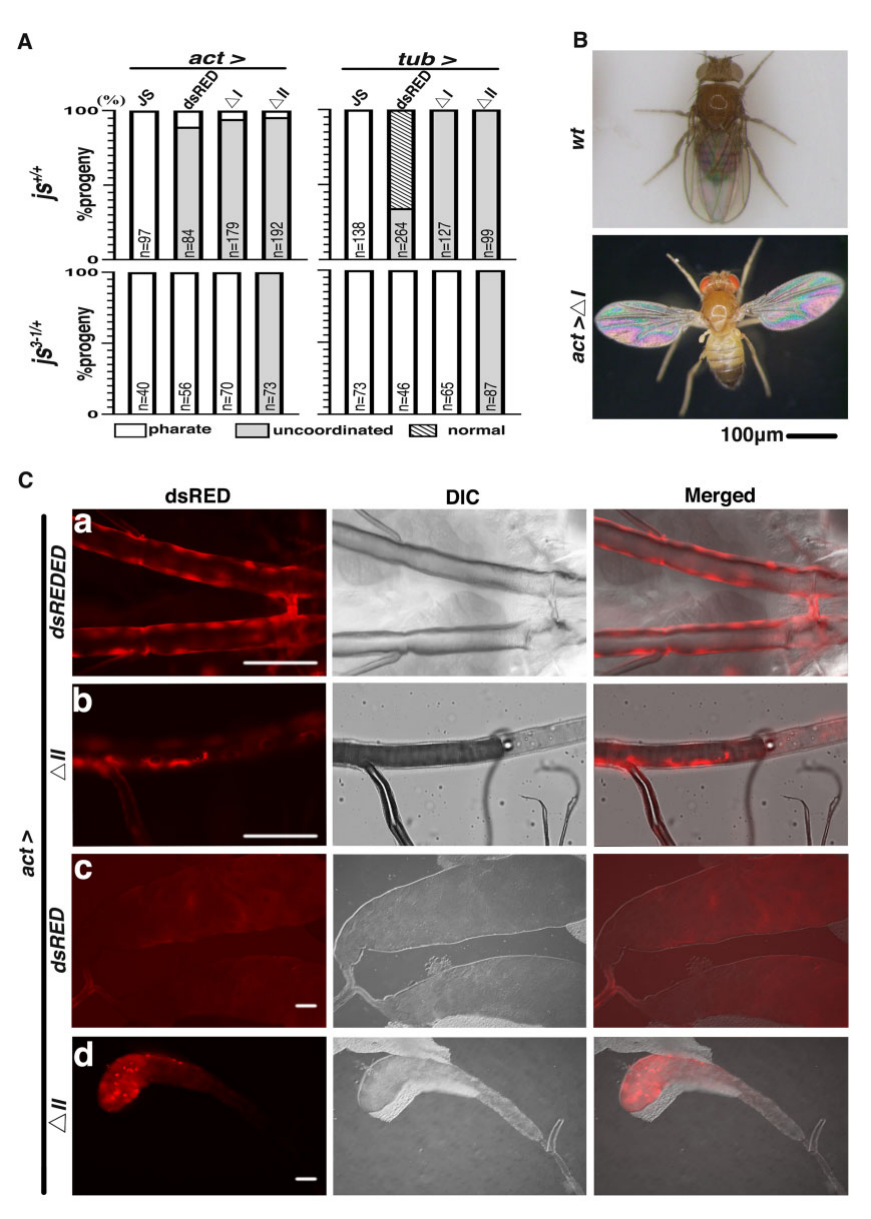
Overexpression of JS disrupts its endogenous function. A) Quantitative analyses of adult phenotypes from JS overexpression. Adults are classified into 3 classes (“pharate,” “uncoordinated,” and “normal”), and their counts are plotted according to different JS over-expressing conditions. The Gal4 driver used is listed at the top with the JS proteins being over-expressed listed underneath. JS: full-length JS protein; dsRED: full-length JS with a C-terminal dsRED tag; ΔI: JS protein with the CBD deletion I and a dsRED tag; ΔII: JS protein with the CBD deletion II and a dsRED tag. The state of the endogenous *js* loci is listed to the left. The number of progenies counted are presented as “*n*” in each column. B) The “spread-wing” phenotype of an adult overexpressing ΔCBD-I with the actin5C driver (bottom). A wild-type adult with a normal wing posture is shown at the top. C) Ectopic localization of dsRED-tagged JS derivatives expressed from the act-Gal4 driver in an otherwise wild-type background. Representative images are shown in triplets: red fluorescence (dsRED), DIC, and the merged image of the 2. Ca and Cb) Larval trachea showing big patches of dsRED fluorescence. Cc) dsRED localization in the main lobes of the larval salivary gland, but missing from the imaginal rings. Cd) aggregation of dsRED signals in secretion cells of the larval salivary gland. Scale bars indicate 100 µm.

In an attempt to “rescue” the js phenotypes, we constructed transgenic lines carrying a *js* cDNA clone under the control of *UAS* elements. We used various Gal4 drivers to deliver JS proteins to the *js*-mutant background. None of the tested Gal4 drivers was able to rescue the lethality of the homozygotes. In addition, we discovered that the strong and ubiquitous Tubulin-Gal4 (tub-Gal4) or Actin5C-Gal4 (act-Gal4) driven overexpression of JS in a wild-type background was sufficient to cause the js phenotypes (Fig. 6A). This effect could also be reproduced by over-expressing a JS-dsRED fusion protein (Fig. 6A). In addition, we constructed 2 separate deletions within the conserved CBD, with deletion I more N-terminal to deletion II (Fig. 2a), and tagged both proteins with a C-terminal dsRED moiety. Both truncated proteins were similarly overexpressed by act-Gal4 or tub-Gal4, and their effects on adult survival were measured.

We classified JS-overexpressing adults into 3 phenotypic classes: pharate or short-lived adults, adults with uncoordinated movements, and normal adults (Fig. 6A). Most flies, dead or alive, had black-pigmented joints on their legs. Under the wild-type (*js^+/+^*) background, act-Gal4 driven production of full-length JS proteins resulted in essentially 100% lethality (top row in Fig. 6A) with most adults dying soon after eclosion and the rest died as pharate adults. Interestingly, when a C-terminally dsRED tagged JS protein was overproduced similarly, a significant portion of the adults survived but many of whom suffered uncoordinated movement suggestive of a *js*-like but milder defect (compare the first and second columns in the top row of Fig. 6A). JS overproduction driven by tub-Gal4 resulted in a similar but milder phenotype than the act-Gal4 driver (top row, left panel of Fig. 6A).

The 2 CBD-truncated JS proteins, when overproduced, also led to physiological consequences. Overproduction of both proteins had a similar effect as JS-dsRED overproduction when act-Gal4 was employed (top row in Fig. 6A). Although overexpression of the 2 classes of dsRED-tagged JS proteins (wild type or CBDdeleted) overwhelmingly yield “uncoordinated” adults, a unique wing phenotype was only produced by the latter. Essentially all surviving flies have their wings spread horizontally (Fig. 6B). The results from tub-Gal4-driven overexpression were largely consistent with ones from act-Gal4, except that overexpressing JS-dsRED had a much milder effect resulting many normal looking adults (top row in Fig. 6A). As all *UAS*-containing constructs were inserted into the same genomic location of 75B via phiC31 integrase mediated insertion, chromosomal position effect on the transgenes must not have been the underlying cause for this phenotypic variation. We suggest that it is more likely caused by the difference in tissue specificities of the 2 Gal4 drivers.

We hypothesized that JS overexpression might have interfered with the normal function of JS leading to phenotypes similar to JS loss of function. We investigated this potential “dominant negative” nature of JS overproduction by repeating it in a *js* heterozygous background, and expected an enhancement of the negative effect of JS overproduction. Our hypothesis is largely supported by the results. As shown in Fig. 6A (compare results in top and bottom rows), adult lethality was driven to essentially 100% with JS-dsRED regardless of which driver was deployed. Even over-expressing JS^ΔCBD-I^-dsRED in a *js*-heterozygous background was sufficient to result in complete lethality. Remarkably, overproduction of JS^ΔCBD-II^-dsRED produced similar phenotypes in the 2 *js* background: no lethality but all uncoordinated. This suggests that the “spread-wing” phenotype is likely the result of a “gain-of-function”, instead of a “dominant negative” effect from overproducing a defective and possibly mis-localized JS protein. Interestingly, ΔCBD-II deletes one of the N-linked glycosylation sites (Fig. 2A), implying the importance of this modification to JS function. In summary, the above results suggest that overexpression of even partially functional JS proteins disrupts the endogenous JS functions, consistent with that JS overexpression exerts a poisoning effect.

### The genetic determinants of JS localization

To provide further supporting evidence that JS overproduction interfered with the normal JS functions, we studied the localization of dsRED-tagged JS proteins ectopically produced from act-Gal4 in tissues using live fluorescence. We discovered that in most of the tissues where we previously observed JS-mCherry signals (at the endogenous level), JS-dsRED is also present but with a much stronger intensity. Some of the examples are shown in Fig. 6C. In particular, the puncta appearance of JS-mCherry molecules changes to a more intense and “sheet” like appearance of JS-dsRED, possibly due to the large amount of JS-dsRED or its mis-localization in the tissues of interest. Interestingly, ectopic JS-dsRED signals were also observed where normal JS-mCherry signals were not discernable, e.g. the secretory cells of the larval salivary glands (Fig. 6C), suggestive of protein mis-localization. The localization of the 2 CBD affected JS proteins are similar with each other but differ mostly from the pattern of the endogenous JS protein. Most prominently, neither is discernable in pericardial cells where endogenous JS is present. In addition, large aggregation of CBD-affected JS-dsRED proteins are present in salivary glands and trachea of the larva. This aggregation is most prominent for the JS^ΔCBD-II^-dsRED protein (Fig. 6C). These results are consistent with that the CBD domain is critical for JS localization. They also support our previous propositions that JS^ΔCBD-II^-dsRED is a defective protein and that overexpression of a normal JS protein is needed to effectively disrupt the function of the endogenous JS.

### Relationship of JS with the previously identified Mucin-D protein

A glycosylated protein named Mucin-D has been extensively characterized by Dr. A. Kramerov and colleagues (e.g. Kramerov et al. 1996, 1997). Several pieces of evidence support the hypothesis that Mucin-D and JS are related, and possibly the same protein. (1) Mucin D was previously purified from various lines of cultured *Drosophila* cells. It was characterized as a secreted glycoprotein, a character shared with JS. (2) Radio-labelled sugar was used to estimate the molecular size of Mucin-D as being between 70 and 100 kDa depending on running conditions of SDS-PAGE. Interestingly, Mucin-D molecules sometimes showed 2 migrating bands around 70 and 100 kDa in size, similar to JS’s behavior on an SDS-PAGE. (3) Remarkably, the determined amino acid compositions of Mucin-D match those of JS very well in that the percentages of different residues between the proteins are correlated with very high significance (Spearman’s test, *r_s_* = 0.8135, *P*(2-tailed) = 7E−05, Supplementary Table 1).

IgM antibodies against Mucin-D using semi-purified native protein as antigens were previously generated. Unfortunately, none of the ones we tested recognizes JS on western blots even when we used fly extracts overexpressing JS. Therefore, whether JS and Mucin-D are the same protein required further investigations.

## Discussion

In this study, we characterized lethal mutants with a novel adult phenotype and identified the affected *js* gene encoding a secreted glycoprotein that bears basic domain features of mammalian Mucins. We also studied the extensive localization of the JS protein in different tissues and developmental stages. Although the exact physiological function of JS remains undetermined, it is likely to be a widely distributed Mucin-like molecules in *Drosophila*, one of the best models for developmental studies with facile genetics. Future investigations into the consequences of both the loss and gain of JS function promise to further our understandings of Mucins in development and disease.

### JS is a new member of the Mucin-like proteins in *Drosophila*

Bioinformatic and limited functional studies have led to the identification of many proteins with signatures of mammalian Mucins in *Drosophila* and other insects (Schwientek et al. 2007; Syed et al. 2008; Dias et al. 2018). Interestingly, JS was not among the previously identified ones, suggestive of the limitation of bioinformatic and genome-wide analyses. In addition to having the most basic features of nonmembrane associated Mucin-like proteins (secreted, glycosylated, having an N- and a C-terminal cysteine-rich domains, and most importantly, having a middle PTS-rich domain), JS has a distribution pattern highly consistent with the general function of Mucin. It is present at the cuticle, digestive, respiratory and reproductive tracts, where the epithelia interact with the environment. In addition, JS is present at internal epithelia in many organs, suggestive of specialized functions, e.g. joints between leg segments, bases at bristles and wing blades, spaces between ommatidia. Therefore, results from our localization studies of JS are consistent with JS serving a similar function as general Mucins in other organisms. In addition, based on a highly correlated amino acid composition between JS and the previously identified Mucin-D protein, we proposed that *js* likely encodes Mucin-D, which Mucin-like properties have been extensively characterized by Kramerov and colleagues. Therefore, JS might represent an excellent example of a general Mucin-like molecule in insects. However, a key piece of information concerning whether JS protein is glycosylated with *N*-acetyl-galactosamine, a modification enriched on mammalian mucins, remains missing.

### Potential functions of JS

The facts that JS’s presence is widespread early in development and that it persists throughout organogenesis seem inconsistent with the late manifestation of physiological defects in mutant adults. In fact, we argued that protein localization that we presented for JS-mCherry in Figs. 3 and 4 is unlikely to reflect the entire JS distribution pattern as we relied entirely on JS-mCherry’s ability to fluoresce under a molecular environment that we have very little knowledge about. One possibility is that defects in *js* mutants are sub-lethal earlier on in development, but rearing conditions under the laboratory settings relax the requirements for JS functions. In other words, more sensitive assays are needed to reveal defects in early *js*-mutants to justify the omni-presence of this protein. For example, the presence of JS in the respiratory and digestive systems might suggest a protective role, the loss of which might not have damaged the luminal surfaces as we confirmed in EM studies. Physical and/or chemical challenges might need to be applied to reveal protective functions that are compromised in the mutants. Moreover, whether the prominent presence of JS in nephrological cells carry functional significance requires further investigation. Alternatively, it might simply reflect that JS is circulating in the hemolymph as excess proteins in the hemolymph are shown to be filtered by the nephrocytes (Weavers et al. 2009; Zhang, Zhao, and Han 2013).

Interestingly, JS proteins are internal to several different body structures, e.g. at spaces between ommatidia, at the bases of bristles, and at the junctions between leg segments. It is plausible that JS serves the function of a lubricant to facilitate movements of body parts, and the lack of lubrication might have been the underlying cause for the breakage of leg joints giving rise to the most distinct phenotypes of *js* adults. Specialized assays would be needed to determine whether lubrication for organ movements is disrupted during earlier development of *js* mutants.

The observation that overexpressing JS or derivatives disrupts the function of the endogenous protein suggests interesting possibilities of JS regulation. First, the presence of excess JS might be sufficient to disrupt the molecular stoichiometry of the mucin barrier. Second, the abnormally produced JS might lack functionally important posttranslational modifications and/or processing as the acting cellular machineries are likely overwhelmed by the mass production of these secreted molecules. A similar effect on protein folding might exist when JS is being overproduced. In sum, improperly processed and/or mis-folded JS molecules might be incorporated into the epithelial networks where JS is a normal component, thus disrupting these networks.

### The future of JS studies

Our JS study lays the groundwork for future structural and functional studies of Mucin-like molecules in a genetic model. In addition, both loss- and gain-of-function tools are available. (1) On the issue of targeted localization, how does JS achieve its precise distribution? As JS has a well conserved CBD (Jasrapuria et al. 2010; Tetreau et al. 2015), it is highly likely that chitin-binding serves as a primary localization mechanism for JS. In fact, the 2 CBD-deleted JS-dsRED derivatives show signs of mis-localization (Fig. 6C), which is consistent with a critical role of chitin-binding in JS localization. However, the Cys-rich N-terminal domain of mammalian Mucins participates in inter- and intramolecular crosslinking. Whether JS’s CBD has a similar function and how the JS–chitin and JS–JS modes of interaction are coordinated would be of future interest. How the conserved CRM at the C-terminus determines JS junction also awaits further investigations. (2) On the issue of posttranslational modifications, how does the extent of JS glycosylation affect its function? The expected O-linked glycosylation sites reside in the PTS-rich domain in the middle of JS. Strategically placed in frame deletions would help identify functionally critical glycosylation positions and scope. In addition, identifying the glycosylase(s) responsible for modifying JS would be of future interest. As most if not all of the enzymes responsible for O-linked glycosylation have been identified in *Drosophila* (Tran et al. 2012; Zhang and Ten Hagen 2019), RNAi knockdown combined with a PAGE-based glycosylation assay would yield important insights. (3) On the issue of JS secretion, what are the major cell types responsible for JS secretion? Do these cells carry similar characteristics of mammalian goblet cells that specialized in Mucin secretion?

## Data availability

Strains and plasmids are available upon request. The authors affirm that all data necessary for confirming the conclusions of the article are present within the article, figures, and tables. Supplemental material is available at figshare: https://doi.org/10.25387/g3.19091528.
